# Nonlinear vocal phenomena and speech intelligibility

**DOI:** 10.1098/rstb.2024.0254

**Published:** 2025-04-03

**Authors:** Andrey Anikin, David Reby, Katarzyna Pisanski

**Affiliations:** ^1^ Division of Cognitive Science, Department of Philosophy, Lund University, Box 192, SE-221 00, Lund, Sweden; ^2^ ENES Bioacoustics Research Laboratory, CRNL, CNRS, Inserm, University of Saint-Etienne, Saint-Etienne 42100, France; ^3^ Institute of Psychology, Institut universitaire de France, Paris, France; ^4^ Institute of Psychology, University of Wrocław, Wrocław 03-815, Poland

**Keywords:** speech, evolution of language, nonlinear vocal phenomena, voice, formant frequencies, vocal membranes

## Abstract

At some point in our evolutionary history, humans lost vocal membranes and air sacs, representing an unexpected simplification of the vocal apparatus relative to other great apes. One hypothesis is that these simplifications represent anatomical adaptations for speech because a simpler larynx provides a suitably stable and tonal vocal source with fewer nonlinear vocal phenomena (NLP). The key assumption that NLP reduce speech intelligibility is indirectly supported by studies of dysphonia, but it has not been experimentally tested. Here, we manipulate NLP in vocal stimuli ranging from single vowels to sentences, showing that the vocal source needs to be stable, but not necessarily tonal, for speech to be readily understood. When the task is to discriminate synthesized monophthong and diphthong vowels, continuous NLP (subharmonics, amplitude modulation and even deterministic chaos) actually improve vowel perception in high-pitched voices, likely because the resulting dense spectrum reveals formant transitions. Rough-sounding voices also remain highly intelligible when continuous NLP are added to recorded words and sentences. In contrast, voicing interruptions and pitch jumps dramatically reduce speech intelligibility, likely by interfering with voicing contrasts and normal intonation. We argue that NLP were not eliminated from the human vocal repertoire as we evolved for speech, but only brought under better control.

This article is part of the theme issue ‘Nonlinear phenomena in vertebrate vocalizations: mechanisms and communicative functions’.

## 1. Introduction

After centuries of intense debate, the question of how humans have evolved language continues to fascinate [1,2]. An important part of the full story is identifying the neurological and anatomical adaptations that are needed for the production of articulated speech. Neurologically, dense projections in the brain from motor cortices to phonatory motor neurons are associated with better vocal control across species [3], and humans stand out as having well-developed, monosynaptic connections between the primary motor cortex and laryngeal motor neurons. These direct connections are thought to be a key adaptation for flexible vocal learning and voluntary control over vocal production, which are prerequisites for speech [4,5]. At the anatomical level, an obvious change in the human vocal apparatus relative to other apes is the modified pharyngeal region of the vocal tract, which places the tongue in a favourable position for articulation, expands the available vowel space and makes articulation more efficient [6–8].

Recent debates have centred on the significance of the simplified human larynx, which lacks vocal membranes and laryngeal air sacs. Air sacs likely function as additional supralaryngeal resonators, whereas vocal membranes function as additional oscillators attached to the vocal folds. Both are found in nonhuman great apes and in many other anthropoid primates, but not in humans [9–11]. These anatomical structures are generally considered to be adaptations for making vocalizations louder or lower in frequency and for increasing the complexity of the vocal repertoire [12–15]. So, why have air sacs and vocal membranes disappeared in the human lineage?

Perhaps these anatomical structures simply became redundant. For instance, Hewitt *et al.* [9] hypothesized that air sacs prevent hyperventilation during intense vocalizing, which was no longer necessary once humans had evolved better breathing control. Alternatively, they may have become too costly. One hypothesis is that air sacs increased the risk of respiratory infections after our ancestors dispersed from Africa, so they were lost and the larynx descended in compensation as an alternative way to produce lower formant frequencies (resonances of the vocal tract, which depend on the length of the vocal tract), and thereby to sound larger [5].

A bold new hypothesis by Nishimura *et al*. [15] ties the anatomical simplification of the human laryngeal region to the need for a stable and tonal vocal source for speech. The authors base this view on their finding that vocal membranes lead to irregular phonation with abundant nonlinear vocal phenomena (NLP). There are several common types of NLP that often result in an unstable or harsh-sounding voice quality, such as frequency jumps (sudden changes in the rate of vocal fold vibration), subharmonics (the appearance of a second, harmonically related frequency), amplitude or frequency modulation and deterministic chaos—possibly the most salient form of NLP. Nishimura *et al*. argue that speech requires a tonal and stable vocal source that is free of these nonlinear phenomena because NLP create ‘an irregular and changing spectrum thought to obscure the conformation of the vocal tract filter … by introducing spurious spectral peaks or “pseudoformants’’ ’ (p. 763). In what follows, we examine this hypothesis, review the relevant evidence and test it experimentally by synthetically adding different types of NLP to speech stimuli.

Acoustically, speech requires some kind of sound source—something that generates the sound itself and that can be turned on and off. The resulting sound also needs to be frequency-modulated to create variable intonation or lexical tones and filtered by the resonances of the vocal tract [16,17]. The source of acoustic excitation during the production of voiced phonemes (e.g. all vowels and voiced consonants like /d w r/) is the nearly periodic vibration of the vocal folds, known as phonation. The rate of vocal fold vibration determines the fundamental frequency $\left(f_{\text{o}}\right)$ of the voice, which we perceive as voice pitch. In voiceless phonemes (e.g. /t s h/) and in whispered speech, the source of sound is aperiodic noise caused by airflow past obstructions [18,19]. While the vocal folds represent the sound source, the vocal tract is the sound filter. Because the vocal tract acts as a resonating chamber, the spectrum of the radiated sound differs from that of the sound source: the frequency bands around each vocal tract resonance become more prominent, giving rise to vocal tract resonances or formants.

Speech is strongly dependent on rapid changes in the shape of the vocal tract achieved with the help of articulators (the tongue, lips, lower jaw and velum), which create distinct patterns and transitions of the first three formant frequencies. The relative spacing between the first few formants is what distinguishes between dozens of different vowels, diphthongs and consonants like /d b g/ [19]. Formant transitions are not the only acoustic features that encode information in speech: temporal modulations, rapid and very precise voicing onsets/offsets and intonation (especially in tonal languages) are also central. Nevertheless, the ability to track formants is a crucial prerequisite for speech intelligibility: synthetic speech that contains nothing but sine waves tracing a few formant contours is still comprehensible [20], whereas speech with artificially removed spectral modulations corresponding to formants is not [21].

Given the centrality of formants to encoding linguistic information in speech, anything that masks formant frequencies is bound to impact speech intelligibility. Undersampling of the transfer function is one common problem: if the harmonics in the source spectrum (integer multiples of the fundamental frequency) are too sparse, vocal tract resonances are not excited and remain inaudible [22,23]. This is not a concern within the normal speech range of humans, in which *f*_o_ does not correlate with speech intelligibility within sexes [24,25]. Indeed, women are typically more intelligible than men despite speaking with a much higher *f*_o_ and thus sparser harmonics [26]. Shouted speech, however, is already less intelligible than spoken speech at the same loudness, partly because shouting increases *f*_o_ [27], and the difficulty is compounded in the range of screams and soprano singing with *f*_o_ that can exceed 2 kHz. Adding some variability to the vocal source, such as vibrato (rapid frequency modulation), may mitigate this problem because harmonics will occasionally cross formants [28]. Nevertheless, there is a limit to how high the voice pitch can be pushed before speech becomes unintelligible.

A related problem is caused by excessive breathiness, which is characterized by a weak and unstable voice with too little energy in harmonics to excite the higher resonances [29,30]. An interesting limiting case is whispered speech, in which the vocal folds are not vibrating at all and there is no harmonic structure or pitch. While obviously intelligible, whispered speech usually shows lower comprehension scores compared with normal modal speech [25,31,32], which is not surprising because it contains no voicing distinctions, nearly halving the number of distinguishable consonants in a language like English. Interestingly, whisperers make other adjustments to their voices to compensate for this and to be better understood [33], and whispered speech shows a number of systematic differences vis-à-vis normal speech, including elevated formant frequencies [31,34,35], increased formant bandwidths [34] and modified spectrum of consonants that partly mimics voicing distinctions [35]. Hypothetically, some types of dysphonia and NLP may similarly affect speech intelligibility owing to obscuring voiced/voiceless contrasts, in which case their impact on speech comprehension should be comparable with that of switching from normal phonation to whispering.

An interesting alternative approach to searching for acoustic changes that degrade speech intelligibility is to ask how speech can be made easier to understand. The speaking style known as *clear speech* tends to have a more variable and higher *f*_o_, with more energy in high frequencies (possibly as a side effect of speaking more loudly [36]), longer phonemes and pauses, clearer syllable articulation, emphasized consonants, an expanded vowel space, clear formant transitions (e.g. in voiced stops), narrower formant bandwidths and fewer vowel reductions [26]. These characteristics are consistent with the notion that formants are central to speech comprehension. However, NLP would not necessarily interfere with any of these elements of *clear speech*. So, what is the evidence behind the hypothesis that NLP would be detrimental for speech intelligibility? This hypothesis rests on three key assumptions.

**Assumption #1**: *Anatomical simplification of the human vocal apparatus has made the vocal source more stable, reducing the probability of NLP*

As noted above, vocal membranes are present in all primates with the exception of humans, and were thus lost at some point in our evolutionary history. Vocal membranes are thin projections on the vocal folds, akin to ‘vocal lips’, that improve vocal efficiency by lowering the phonation threshold pressure. When chimpanzees vocalize, their vocal membranes are engaged during the production of both high-pitched screams and low-pitched grunts [15]. Combined with physiological data from monkeys, there is some evidence that vocal membranes—and not the vocal folds—represent the main vocal source in nonhuman primates [15]. Possibly as a result of this anatomy, vocalizations of many primates tend to be noisy and easily slip into NLP, especially in loud vocalizations.

In general, systems with more oscillators are capable of more complex dynamics and prone to sudden transitions (bifurcations) between different vibratory modes [13,37]. Both biomechanical models and experiments with silicone replicas of vocal folds have now shown that additional oscillators, such as vocal membranes and ventricular folds (i.e. a second set of vocal folds, which humans do have), indeed increase the occurrence of NLP [12–15]. Some modelling also suggests that laryngeal air sacs predispose to irregular phonation because they make the filter more complex and introduce additional low resonance frequencies [38,39], making *f*_o_ more likely to cross formants and therefore potentially increasing the probability of NLP owing to source–filter interaction [40–43]. Thus, anatomical simplification of the vocal apparatus in modern humans has probably reduced acoustic complexity of an involuntary kind such as spontaneous frequency jumps, voice register changes and transitions between tonal phonation and various NLP. Importantly, however, NLP have been extensively documented in healthy humans, including in singing [44–46], nonverbal vocalizations [47–49] and speech [50]. NLP can occur without involving other oscillators than the vocal folds, but humans have also retained some supralaryngeal structures capable of sustained vibration and implicated in causing NLP—notably the ventricular and aryepiglottic folds [13,37,46,51,52]. Thus, the simplification of the human vocal apparatus is incomplete, and the vocal source in anatomically modern humans is by no means uniformly stable and is still capable of producing a variety of NLP.

**Assumption #2**: *Vocal instability impacts speech intelligibility*

The best-supporting evidence for this comes from clinical studies of dysphonia. Patients with adductor spasmodic dysphonia, which leads to a strained, harsh, monotonous voice with voice stoppages and breaks, are easier to understand after an injection of the botulinum toxin (botox), which blocks the twitching muscles [53]. More generally, numerous clinical and experimental studies of voice pathology have shown that dysphonia affects speech intelligibility [24,29,54–57]. However, clinical definitions of dysphonia do not specifically differentiate between different types of NLP and a variety of other vocal abnormalities such as tremor (a ‘shaky’ voice), breathiness, voice stoppages and breaks, noisy phonation, etc. Studies of dysphonia also tend to be correlational, with very few tested speakers [24,29,54–56], or rely on healthy speakers who simply imitate dysphonia [57,58]. Thus, it is not possible to conclude whether or not NLP, let alone specific NLP types, causally contribute to the reported loss of intelligibility based on the dysphonia literature. Testing this hypothesis directly and experimentally is thus a key aim of the present study.

**Assumption #3**: *NLP obscure the formant structure*

In their original publication, Nishimura *et al.* [15] cite three studies in support of this view. One of these [59] established that rhesus macaques notice formant shifts in noisy calls, which actually seems to work against their hypothesis as it indicates that noise does not obscure formants. The other two studies showed that tonal calls were better than noisy grunts and screams at conveying individual identity in chacma baboons [60] and rhesus macaques [61], wherein information about caller identity is often encoded in formants. However, screams are much more high-pitched than the tested tonal calls, while grunts are shorter in duration, so these correlational data cannot tell us whether NLP are responsible for the observed differences.

We are not aware of other empirical evidence to support Assumption #3. To evaluate it theoretically, let us consider specific NLP types. Subharmonics are characterized by the appearance of a secondary, lower frequency at a rational fraction of *f*_o_, often *f*_o_/2 or *f*_o_/3. Perceptually, this sounds like two harmonically related tones produced simultaneously. Looking at a spectrogram, subharmonics increase the density of partials in the original spectrum without changing its basic structure; it is not obvious how this could obscure the vocal tract conformation. Biphonation and low-frequency amplitude modulation (AM) are basically similar to subharmonics in introducing a secondary frequency that modulates the main vocal source, but can change independently of *f*_o_. This situation often produces sidebands around each harmonic on a spectrogram, and these sidebands can indeed look like pseudo-formants on a spectrogram [23]. Deterministic chaos makes the spectrum noisy, and the distorted harmonics in high-pitched vocalizations are easily mistaken for formants on spectrograms [23]. As a result, it is often assumed that chaos would make vowels difficult to recognize [62], but again we are not aware of any perceptual data to confirm this.

A possible counter-argument is that subharmonics, sidebands and chaos might instead highlight rather than obscure the formant structure because a dense source spectrum better reveals the transfer function of the vocal tract [39,63], just as formant perception becomes easier if *f*_o_ is lowered [22,64]. In other words, nonlinear phenomena lead to a denser ‘audio picture’ not unlike a photo with more pixels, potentially making formants easier to detect. Both positive and negative effects of NLP on formant perception could also occur simultaneously, and they may vary by NLP type or depending on other acoustic qualities of the vocal signal. For example, the net effect could be beneficial in high-pitched shouts or screams with very sparse harmonic spectra, but detrimental in low-pitched vocalizations such as ordinary speech.

In this study, we performed three psychoacoustic experiments to test Assumptions #2 and #3 empirically. To do so, we investigated the effect of target NLP manipulations on speech intelligibility at the level of individual phonemes, words and short phrases. We began by testing whether the presence of NLP would highlight or mask formant transitions in isolated synthetic vowels (Experiment 1). We then tested whether listeners could understand the sequences of unconnected words (numbers) with various forms of manipulated NLP (Experiment 2). This placed NLP in a more complex and ecologically valid phonetic context, but still largely avoided linguistic effects related to violations of normal prosody. Finally, we manipulated various forms of NLP in short sentences that listeners needed to transcribe in easy or challenging listening conditions, depending on the presence of masking noise (Experiment 3).

## 2. Methods

### (a) Experiment 1: Synthetic diphthongs

#### (i) Stimuli

The stimuli used in Experiment 1 (*n* = 4995) were isolated synthetic vowels or diphthongs, each 700 ms in duration and with all acoustic energy in harmonics (i.e. without any turbulent noise component). Five basic vowel types were taken from the library of the *soundgen* package in R [65]: /a o e i u/. The values of the first four formants (F1 to F4) in these vowels corresponded to those measured in a single male speaker, and formants above F4 were added automatically up to the Nyquist frequency based on the estimated average formant spacing. Half the stimuli were monophthongs with completely static formants corresponding to one of these five vowels /a o e i u/, and the other half were diphthongs starting at one vowel and morphing linearly into another over 700 ms, either all the way or partially, with morphing steps of 10% (0%: monophthong; 100%: full diphthong). The *f*_o_ of each vowel was chosen stochastically using a uniform distribution on a logarithmic scale between 110 Hz (musical note A2) and 880 Hz (A5).

**Figure 1.**
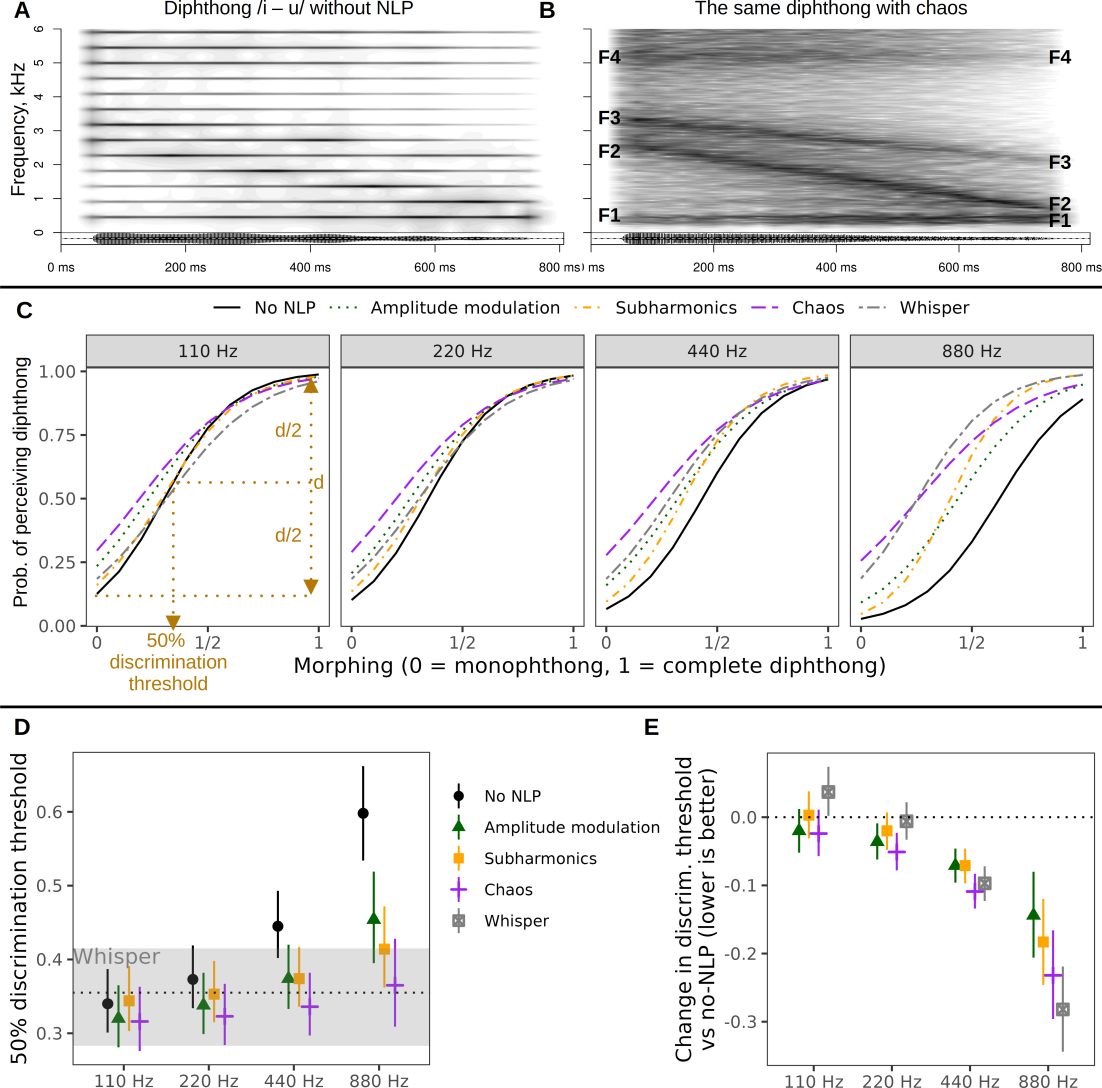
NLP improve vowel perception at high pitch. (A,B) The creation of synthetic vowels: an example of a full diphthong /i – u/ with *f*_o_ = 454 Hz (musical note A♯4). Narrow-band spectrograms with a 50 ms Gaussian window are shown. The formants (F1–F4) are much less obvious in the condition without NLP (A) compared to the condition with chaos (B) because chaos provides a denser spectrum. (C) Listeners are more likely to perceive a diphthong as the amount of vowel morphing (from monophthong to diphthong) increases, but without NLP, vowel discrimination becomes very hard at high pitch. Fitted values from multilevel logistic regression; effects are shown for four pitch values ranging from 110 Hz (musical note A2) to 880 Hz (A5). 50% discrimination thresholds are calculated for each curve as the amount of morphing that raised the probability of perceiving a diphthong halfway from floor (morph = 0, pure monophthong) to ceiling (morph = 1, complete diphthong). (D) These discrimination thresholds are similar for all conditions at lower pitch, but increase at higher pitch, particularly for the *no-NLP* condition. The grey shaded area shows the discrimination threshold for the *whisper* condition, with 95% CI. (E) NLP dramatically lower discrimination thresholds and improve vowel perception at high pitch compared to the *no-NLP* condition. All shown estimates are medians of posterior distribution and 95% CIs. *n* = 18 091 classifications of 4879 synthetic vowels by 91 listeners.

We created 999 prototypes with different *f*_o_ values. There was a 50% chance of a prototype being a diphthong with a randomly chosen amount of morphing (*n* = 480) and a 50% chance of it being a monophthong (*n* = 519; 480 + 519 = 999). Each prototype was synthesized in five NLP conditions (*tonal, AM, subharmonics, chaos and whisper*), for a total of 4995 stimuli. Frequency jumps were not tested in this experiment because listeners in pilot tests easily confused changes in *f*_o_ with changes in vowel quality. In the *tonal* condition, only *f*_o_ and its harmonics were synthesized with a ‘rolloff’ value of 3 dB per octave. In the *AM* condition, all synthesis parameters were identical except for the addition of static, unvarying AM with a frequency randomly chosen between 50 and 150 Hz and a depth of between 40% and 60%. In the *subharmonic* condition, period doubling was created throughout the vowel at a steady depth between 40% and 60%. In the *chaos* condition, an imitation of deterministic chaos was created by adding very rapid (1 kHz) and strong (2–4 semitones) jitter, again at the same level throughout the sound. Finally, in the *whisper* condition, the voice source was aperiodic turbulent noise with no harmonic component. The above-mentioned random variation in the characteristics of NLP manipulations across prototypes ensured that the results would generalize to a wide, representative range of possible vocal nonlinearities rather than, for example, a single modulation frequency or a specific amount of chaos. To avoid large changes in sound intensity in diphthongs, a compressor effect was applied to each synthesized vowel using Hilbert envelopes and a 50 ms smoothing window (figure 1A).

#### (ii) Procedure

The perceptual experiment was written inJavaScript and performed online. Participants were asked to wear headphones during the experiment, to adjust the volume at the beginning after listening to a few labelled examples of mono- and diphthong vowels, and not to make further adjustments during the experiment. After providing basic demographics and informed consent, listeners performed 200 trials in about 15 min, each with a new stimulus prototype with a randomly chosen NLP condition. Listeners could replay each stimulus an unlimited number of times to decide whether it was a monophthong (explained as ‘*a single steady vowel*’) or a diphthong (explained as ‘*any variation / change in this vowel (e.g. a diphthong like /a-u/*)’). The response button flashed green for correct answers and red for incorrect answers. An accuracy gauge was shown on the screen and updated after each trial. In addition, after every 50 trials a pop-up window alerted participants whenever their accuracy dropped below the target of at least 65%.

#### (iii) Participants

Participants were recruited on the Prolific platform (www.prolific.com). One was excluded because of completing fewer than 50 trials, and another 13 participants were excluded because their accuracy was below target (65%) even in perceptually obvious conditions (complete diphthongs or monophthongs), suggesting that these participants either could not hear the stimuli properly or did not understand the instructions and responded at random. The average accuracy per person among the remaining 91 participants (47 females and 44 males) was high: median = 78%, range 63–93%. A sensitivity analysis confirmed that the main results remained substantively unchanged when no participants were excluded (electronic supplementary material, figure S1). The mean age ± s.d. was 38 ± 13 years, range 21–79. All participants reported being fluent in English and not having any hearing problems.

#### (iv) Data analysis

All models in all experiments were fit with the *brms* package in R [66] using mildly informative conservative priors. All reported effects are fitted values of model parameters and contrasts between conditions expressed as medians of posterior distributions and 95% credible intervals (CIs). The response was coded as a binary variable (diphthong versus monophthong) and modelled with multilevel logistic regression as a function of NLP condition (a factor with 5 levels), the amount of morphing (a continuous variable ranging from 0 to 1) and voice pitch (a continuous variable ranging from 0.11 to 0.88 kHz). Because some vowels may sound more similar to each other and thus make diphthongs less obvious (especially for the /i – e/ pair), the effect of morphing was allowed to vary across vowels. We also included a group-level intercept for each subject and stimulus:


diphthong∼condition∗morph∗pitch+(morph|vowel)+(1|subject+stimulus)

### (b) Experiment 2: Word discrimination

#### (i) Stimuli

The word stimuli in Experiment 2 consisted of 1300 resynthesized sequences of six digits each, ranging from 2.6 to 4.4 s in duration (mean = 3.6, s.d. = 0.3). The original numbers (1 to 9; zero was not included) were spoken by eight random computer-generated voices: four males and four females. Contours of *f*_o_, spectral envelopes (smoothed with a frequency window of 300 Hz) and amplitude envelopes (smoothed with a time window of 200 ms) of these recordings were extracted and used for resynthesis with the R package *soundgen* [65]. To manipulate NLP, the voice source of each recording (harmonics and noise) was first synthesized using the original *f*_o_ contours with the appropriate modifications for each NLP condition but without any formants. The measured *f*_o_ values were interpolated to smooth contours at the target sampling frequency of 22 050 Hz and the vocal source was created as a mixture of continuous sine waves, one for each harmonic with a realistic rolloff of 9 dB/octave above *f*_o_ and white noise at −30 dB relative to the harmonic component, as described in *soundgen* documentation and in [65]. The original spectral and amplitude envelopes were then smoothed and copied onto the synthetic sources using the *transplantFormants* and *transplantEnv* functions from *soundgen* (electronic supplementary material, figure S2) [67]. Six digits were chosen randomly from the same speaker and concatenated with pauses of 50–200 ms. Masking noise was created once for each six-digit sequence with a flat spectrum up to 1.2 kHz and a spectral slope of −6 dB/kHz above 1.2 kHz using the *generateNoise* function from *soundgen*. This works by setting up the required noise profile in the frequency domain and converting it to the time domain with inverse short-time Fourier transform. The resynthesized numbers and noise were mixed following normalization at a signal-to-noise ratio (SNR) ranging from −6 to 15 dB to make the task moderately difficult and avoid floor or ceiling effects.

The source for each of 1300 sequences was synthesized in a randomly chosen experimental condition: six NLP conditions (*tonal, frequency jump, AM, subharmonics, chaos* or *whisper*) crossed with three pitch levels (*original, +1 octave* and +*1.5 octaves*), for a total of 6 × 3 = 18 combinations. All six digits in a sequence were created in the same condition (same NLP manipulation) and NLP affected the entire duration of each digit (except for frequency jumps, which are acute). In the *frequency jump* condition, a discontinuity point was randomly chosen between 25% and 75% of each digit’s duration, and after this point the remaining *f*_o_ contour was shifted either up or down by a random amount between 3 and 12 semitones. *AM* was added at a frequency between 50 and 150 Hz and a modulation depth of 30–60%. *Subharmonics* were always added at *f*_o_/2 with a depth of 30–50%. *Chaos* was emulated by adding rapid jitter with a depth of 1–4 semitones. The *whispered* condition corresponded to having only white noise as the voice source, with no harmonic component. Except for the *whispered* condition, the entire *f*_o_ contour could also be shifted upwards by 1 octave or 1.5 octaves.

#### (ii) Procedure

Participants heard 100 six-digit sequences and had to enter the six numbers they heard using a physical or virtual keyboard. The experiment was self-paced and there was no time limit per trial, but each sequence was played only once. Participants could start typing as soon as a trial commenced, so there was normally no need to hold the sequences in working memory for longer than it took to type in the numbers. The median trial duration was 6.3 s and the entire experiment with 100 trials took about 15 min.

#### (iii) Participants

We recruited 220 participants (116 females, 100 males and 4 unspecified/other), all of whom completed at least 20 trials (normally 100). The mean age ± s.d. was 31.1 ± 10.5 years, range 18–72. The accuracy per person was high (median = 79.7%, range 47.2–95.0%) and only three participants were excluded because of low accuracy (under 40%). All participants reported being fluent in English and not having any hearing problems. With this sample size, each of 1300 sequences was tested on average in 16.7 trials.

#### (iv) Data analysis

Speech comprehension was operationalized as the match between the target six-digit sequence in each trial and the six-digit sequence entered by the participant, as well as the response time (RT) per trial. Target and input sequences were matched in two ways: (i) by simply comparing each of the six digits and counting errors (ranging from 0/6 to 6/6); or (ii) by performing a dynamic time warp (DTW) and taking the normalized distance between the two sequences. DTW penalizes random errors or inversions more than insertions or deletions, but the difference between DTW and simple matching was small (Pearson’s *r* = 0.84) as the target sequences were relatively short. We therefore focused on simple matching (see electronic supplementary material, figure S3 for DTW and RT analyses).

To model these three measures (simple matching, DTW distance and RT), three Bayesian multilevel models were fit with the *brms* package in R [66]. The model formula was as follows:


{response}∼condition+pitch+s(SNR,by=condition)+(1|sound+subject+speaker),

where *response* stands for the outcome variable, *condition* is a factor encoding NLP manipulation (6 levels: *no NLP, frequency jumps, AM, subharmonics, chaos* and *whisper*), *pitch* is a factor encoding pitch manipulation (3 levels: *original, +1 octave* and *+1.5 octaves*), *SNR* is signal-to-noise manipulation (−6 to 15 dB), *sound* is the identity of a unique sequence (1300 levels), *subject* is the identity of a research participant (219 levels) and *speaker* is the identity of a computer-generated speaker voice (8 levels). The effect of SNR was clearly nonlinear and therefore it was modelled with a smooth term including an interaction with NLP condition. The outcome variable and its distribution were as follows: (i) the number of correct digits out of six for simple matching (binomial); (ii) normalized DTW distance between sequences for DTW error (hurdle-lognormal, with the same predictors used for the hurdle part); and (iii) RT adjusted for variability in the duration of six-digit sequences by subtracting stimulus duration (lognormal).

### (c) Experiment 3: Sentence transcription

#### (i) Stimuli

**Figure 2.**
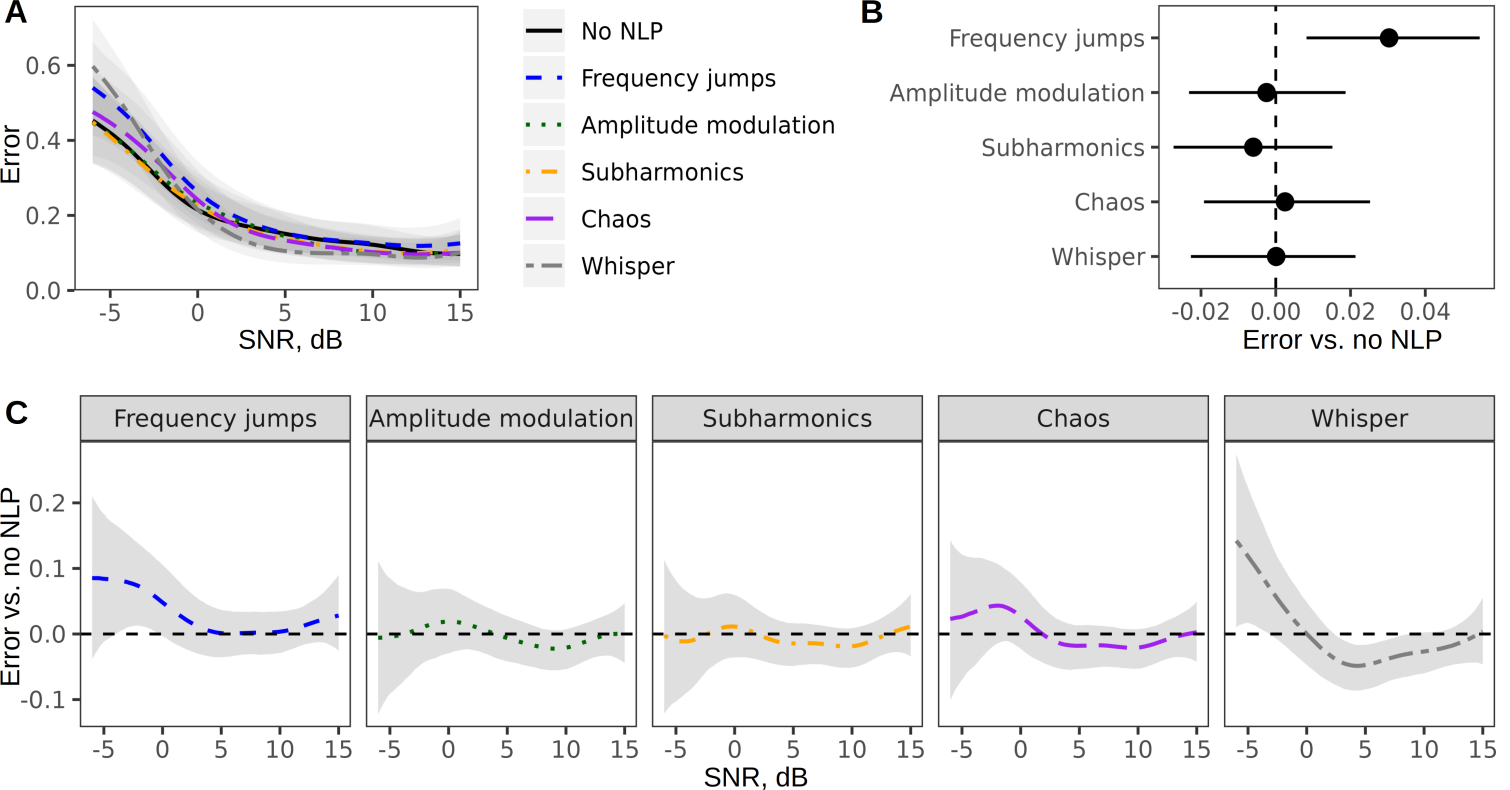
NLP manipulations have little or no effect on the intelligibility of single words. (A) Fewer errors in word discrimination are made as SNR improves, largely regardless of NLP condition. (B) Only frequency jumps slightly increase the number of errors overall. This graphic shows the main effect of NLP condition compared with tonal speech with no NLP (dotted vertical line), averaging across all three pitch levels and all SNRs. (C) Any effect of frequency jumps and whispering on word discrimination is only noticeable at the lowest SNRs, representing the noisiest stimuli and hardest listening conditions. The graphic shows contrasts for each NLP condition versus tonal speech. All estimates are medians of posterior distributions and 95% CIs. Sample size: 1300 six-digit sequences played back to 220 listeners in 21 485 trials.

Target sentences in Experiment 3 were taken from the Harvard IEEE corpus [68], which is often used for studying speech intelligibility [30,69,70]. We used 100 sentences recorded by a female British speaker and ranging in duration from 1.8 to 3.5 s. These were all single-clause sentences such as '*The jacket hung on the back of the wide chair*'*,* '*At that high level the air is pure', 'Drop the two when you add the figures'*, etc. The method of resynthesis was similar to Experiment 2: *f*_o_ contours of each sentence were extracted with careful manual verification of voice onsets/offsets, and the resynthesized vocal source (with harmonic and noise components) was combined with smoothed amplitude and spectral envelopes taken from the original. NLP manipulations were slightly different from those in Experiment 2 because the stimuli were now much longer (sentences rather than words). About five frequency jumps per sentence were added stochastically, with magnitude ranging from 0.5 to 12 semitones (mean ± s.d.: 3 ± 6). The direction of jump was chosen by an algorithm that favoured alternating jumps up and down to avoid producing extreme *f*_o_ values. Other NLP were added either to the entire sentence (the *continuous* condition) or stochastically, in about five separate episodes per sentence (the *intermittent* condition). In the *mixed* condition, frequency jumps and other NLP were chosen and added at random (figure 2A).

#### (ii) Procedure

The perceptual experiment lasted about 20 min and was performed online. Participants were presented with 60 sentences randomly sampled from 100 prototypes and divided into four blocks of 15 trials, with pauses in between blocks. All stimuli within a block were taken from the same NLP condition to habituate participants to each type of voice quality and avoid a novelty effect in each new trial. The same sentence never recurred in multiple blocks. Each stimulus was played once, and listeners were asked to type what they heard in a response box, with no time restriction. The intelligibility of both original and resynthesized stimuli was close to ceiling. We therefore repeated the experiment after adding background noise at a SNR of 0 dB. We used a few seconds of the same continuous and loud street noise as a mask for all recordings.

#### (iii) Participants

Two independent samples of native speakers of British English (matching the language and nationality of the speaker) were recruited to test the stimuli with or without masking noise. Without noise, there were 104 participants (60 females and 44 males; mean age ± s.d.: 39.0 ± 13.1 years, range 18–68). With noise, there were 99 participants (47 females, 51 males, 1 unspecified/other; mean age ± s.d.: 39.9 ± 11.2 years, range 19–63). One participant provided low-effort responses (single words instead of entire sentences) and was excluded from the analysis. With these sample sizes, each of 1200 stimuli was presented ~5 times in each noise condition.

#### (iv) Data analysis

For analysis, the strings of words entered by participants were stripped of extra spaces and non-alphanumeric characters (e.g. punctuation), converted to lower case and then compared with the target sentences. The accuracy of responses was assessed using three distance metrics: WER, Levenshtein distance and Jaro distance. WER has long been the standard metric in evaluating speech recognition [71]. It shows the proportion of words in the target sentence that are correctly understood by the listener, but cannot distinguish between near-hits and complete misses because accuracy is calculated per word. We therefore also calculated two string-based distance metrics. Levenshtein distance is the minimum number of single-character edits (insertions, deletions or substitutions) needed to make two strings identical, normalized by dividing it by the number of characters in the target sentence. Jaro distance is another popular metric that focuses only on the matching characters and allows for transpositions. These three measures were strongly correlated (Pearson’s *r* = 0.88 or higher) and produced very similar results in terms of comparing NLP conditions, so we only focus on WER in the main text (see electronic supplementary material, figure S4 for Levenshtein and Jaro distances). The accuracy (1 – WER) was bounded between 0 and 1 and modelled with a zero-one-inflated beta distribution. We used the same model formula for the mean, zero-one-inflation (zo) and conditional one-inflation (coi) parameters, as follows:


{accuracy,zoi,coi}∼condition+(1|sentence+stimulus+subject),

where *condition* was a factor with 12 levels encoding NLP manipulation.

## 3. Results

### (a) Experiment 1: Synthetic diphthongs

In this first experiment, we tested whether listeners could distinguish between synthetic monophthongs (single vowels) and diphthongs (gliding vowels) when we added various types of NLP (see §2, figure 1A,B). Diphthongs are also known as gliding vowels because they include two adjacent vowels that ‘glide’ into one another in a single syllable, such as /au/, which listeners can readily perceive in normal listening conditions by relying on audible changes in formants. Here, when vowels were synthesized with a low *f*_o_, the presence of NLP had little effect on the listeners’ ability to distinguish between monophthongs and diphthongs (figure 1C, panels with pitches of 110 and 220 Hz). The only impact of NLP was the slightly increased propensity to mistakenly perceive some vowel variation in monophthongs. Compared with the *no-NLP* condition at *f*_o_ = 110 Hz, this ‘diphthong bias’ was +11% (95% CI [7, 15]) for AM, +17% [[12, 22]] for chaos, +6% [2, 10] for whisper and +3% [0, 7] for subharmonics. As the amount of vowel morphing increased and approached a full diphthong, discrimination became essentially perfect in all conditions.

When we synthesized vowels with a high *f*_o_, here tested up to 880 Hz (musical note A5), the discriminability of mono- and diphthongs dropped dramatically in the *no-NLP* condition (figure 1D). However, the presence of NLP *improved* rather than impeded performance: all tested NLP types largely prevented the elevated *f*_o_ from masking formant transitions (figure 1E). At the highest tested pitch of 880 Hz, diphthongs with versus without NLP had much lower discrimination thresholds: AM by 14% [8, 21], subharmonics 18% [12, 25], chaos 23% [17, 30] and whisper 28% [22, 34]. Interestingly, AM and subharmonics did not cause a noticeable ‘diphthong bias’ at very high *f*_o_ (only +2% [0, 4] and 6% [4, 10], respectively, at *f*_o_ = 880 Hz and morph = 0), whereas the *whisper* and *chaos* manipulations were more likely to create a misleading perception of change in a pure vowel (+16% [11, 22] and 23% [17, 30], respectively; see figure 1C).

In sum, the experimentally manipulated NLP were largely irrelevant to the listeners’ ability to perceive formant transitions in synthetic vowels at *f*_o_ levels commonly found in male and female speech (~100 to 300 Hz). At higher *f*_o_ levels typical of shouted speech or screams (~400 to 900 Hz), NLP helped to highlight the formant transitions that would otherwise be very difficult to perceive (figure 1A,B). Subharmonics and AM were particularly effective in this regard because they greatly improved diphthong perception without causing spurious perceptual changes in vowel quality in monophthongs.

### (b) Experiment 2: Word discrimination

Although NLP were found to help with formant perception in isolated synthetic vowels, their impact on the processing of more complex linguistic stimuli may be affected by other factors. To test this, we began by manipulating NLP in single words—namely six-digit strings of numbers that were embedded in masking noise to make the listening task harder. Masking noise is commonly added to stimuli in research on speech intelligibility to avoid ceiling effects and to amplify the effects of manipulations: humans are so good at guessing and filling in gaps that degraded speech is hard to understand particularly [57], or sometimes only [58,72], in noisy listening conditions. In this task, listeners simply needed to identify which numbers they heard (§2).

As expected, our results showed that SNR had a major effect on performance: the numbers entered by participants better-matched target sequences when the signal was less noisy (higher SNR), but comparable SNR–response curves were obtained for all NLP conditions (figure 2A). Looking at the main effect of NLP condition across all SNRs and pitch levels, only the addition of frequency jumps led to a small, but statistically robust increase in errors compared to the condition without NLP: +0.03 (95% CI [0.01, 0.05]; figure 2B). This was clearly driven by the noisiest recordings with SNR < 0 dB (figure 2C). Averaging over SNRs of −6 to −1 dB, the addition of frequency jumps relative to no NLP increased matching errors by +0.07 [0.02, 0.14], whereas no such effect was apparent at SNR > 0 dB: +0.01 [−0.01, 0.03]. Interestingly, the *whisper* condition actually improved performance by +0.03 [0.01, 0.05] at moderate noise levels (SNR > 0 dB), but had the opposite effect in noisy conditions (SNR < 0 dB: −0.07 [−0.13, −0.01]).

Thus, the listeners’ ability to discriminate between a few target words was largely unaffected by NLP, except for a small deleterious effect of adding frequency jumps or unvoicing the sound source in the hardest listening conditions. With only nine possible targets (digits 1 to 9), a few minimal acoustic cues would suffice to distinguish between them. It is therefore possible that NLP did obscure some subtle phonetic features, but these were not essential for comprehension and failed to affect performance. Thus, we next made the task more difficult, open-ended and similar to everyday verbal communication in Experiment 3 by testing entire sentences instead of single words.

### (c) Experiment 3: Sentence transcription

**Figure 3.**
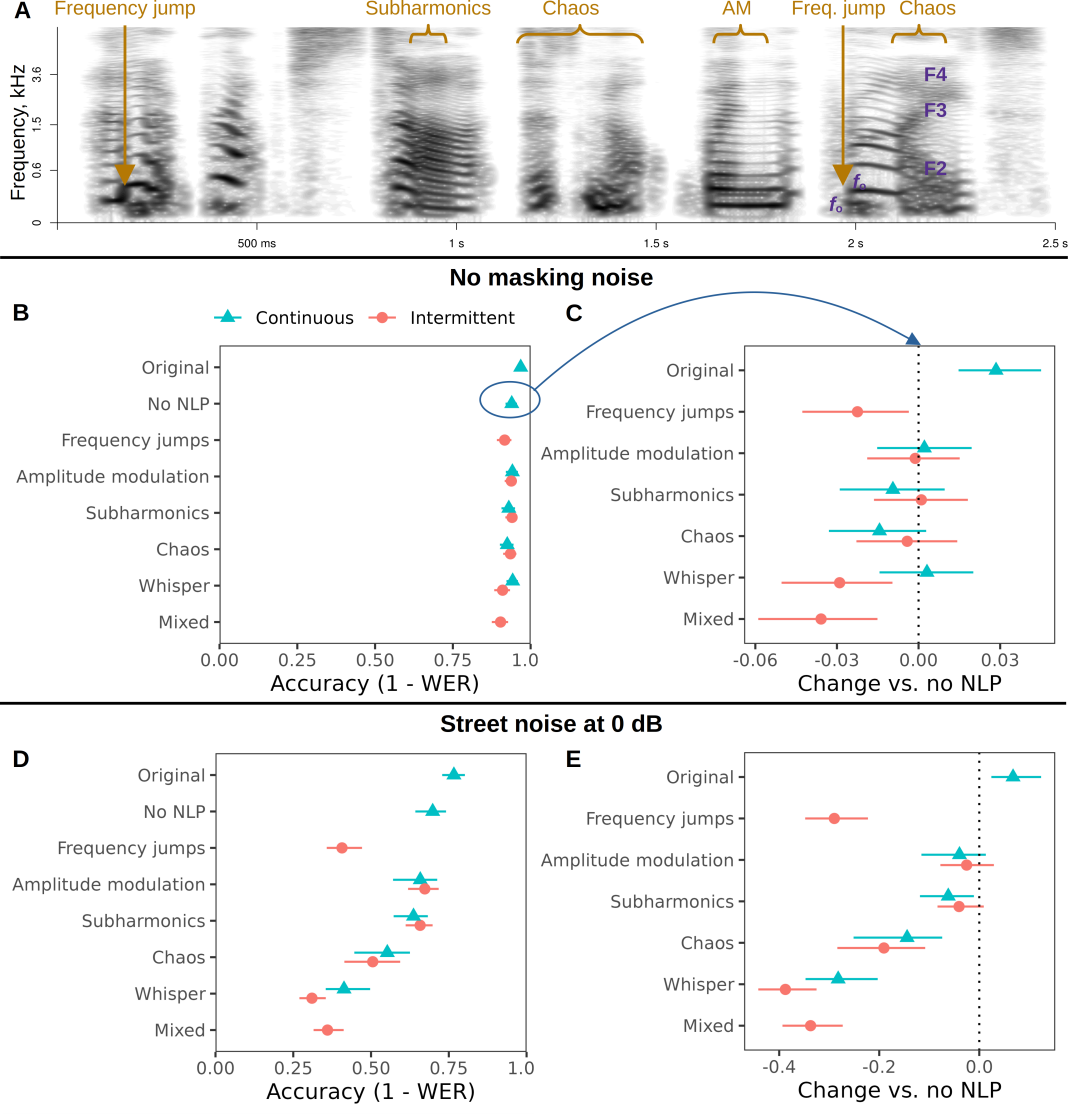
The effect of NLP on the intelligibility of short sentences is absent in normal listening conditions and limited in noisy conditions, depending on NLP type. (A) A spectrogram illustrating the *mixed* condition: a female speaker saying ‘*An abrupt start does not win the prize’*. (B,D) Intelligibility is nearly perfect without masking noise and drops if noise is added: the graphic shows fitted values of word-level accuracy (the inverse of word error rate (WER)) per condition. (C,E) Any drop in intelligibility owing to NLP manipulations appears to be related to instability (*frequency jumps, mixed*) or loss of voiced–voiceless distinctions (*whisper, chaos*). Shown: contrasts with the *no-NLP* condition (negative values indicate worse performance). All estimates are medians of posterior distributions and 95% CIs. *Continuous* = constant voice quality (e.g. with subharmonics) throughout the target sentence; *intermittent* = multiple NLP episodes within one sentence. Sample sizes: 1198 stimuli transcribed by 104 listeners in 6199 trials without masking noise; 1195 stimuli transcribed by 99 listeners in 5811 trials with masking noise.

In this experiment, listeners were asked to transcribe short sentences after listening to them once, with or without loud masking noise and with similar NLP manipulations to those used in Experiment 2 (figure 3A). As expected, strong masking noise caused a considerable drop in performance: global average word accuracy decreased from 90% to 55% and the proportion of perfectly transcribed sentences dropped from 63% to 18% (figure 3B,D), indicating that noise made it much harder for listeners to understand the sentences.

We focused on the effect of NLP manipulations in these two tasks of varying difficulty. To test whether bifurcations were particularly detrimental for speech intelligibility, NLP were added either throughout a sentence (the *continuous* condition) or intermittently, in several shorter episodes (the *intermittent* condition). Because artefacts of resynthesis are more likely when adding NLP to entire sentences, we tested both the original recordings and their resynthesized versions without any NLP as control conditions. Indeed, there was a small loss of intelligibility associated with resynthesis even without adding any NLP: 2.8% [1.5, 4.5] without masking noise and 6.8% [2.4, 12.4] with masking noise.

Using this resynthesized *no-NLP* condition as the new performance benchmark, the addition of *amplitude modulation*, *subharmonics* or *chaos* had no effect on intelligibility in easy listening conditions without masking street noise (figure 3C). The *whisper* manipulation had a small negative effect when it was intermittent (−2.9% [−5.0, −1.0]), and so did *frequency jumps* (−2.2% [−4.3, −0.4]) and the *mixed* condition, in which both frequency jumps and various NLP episodes were added together in a single voice stimulus at random (−3.6% [−5.9, −1.5]). With masking noise, *amplitude modulation* and *subharmonics* still failed to affect intelligibility, regardless of whether they were continuous or intermittent (figure 3E). Continuous *subharmonics* had a negative effect (−6.2% [−11.9, −1.1]), but it was small compared with the massive drop in performance caused by *frequency jumps* (−29% [−34.8, −22.3]) and *whispering* (continuous: −28.2% [−34.7, −20.3]; intermittent: −38.8% [−44.2, −32.5]). The *mixed* manipulation also caused a marked drop in speech intelligibility: −33.7% [−39.3, −27.3]. The *chaos* conditions were intermediate in their effects (continuous: −14.4% [−25.1, −7.4]; intermittent: −19.1% [−28.4, −10.8]).

Contrary to predictions, we did not find any robust difference between continuous and intermittent *amplitude modulation* (a difference of +0.00 [−0.01, 0.02] without masking noise, and −0.01 [−0.09, 0.04] with masking noise), *subharmonics* (−0.01 [−0.03, 0.01] and −0.02 [−0.08, 0.02]) or *chaos* (−0.01 [−0.03, 0.01] and +0.04 [−0.06, 0.14]). However, intermittent *whispering* was indeed worse for intelligibility than unvoicing the entire sentence (by 0.03 [0.01, 0.05] without noise and 0.10 [0.05, 0.18] with noise), presumably because it was confusing to switch between normal and whispered phonation repeatedly within the same sentence or word.

To summarize, none of the tested NLP manipulations compromised speech intelligibility in normal listening conditions, whereas in challenging conditions with added street noise the strongest impact was caused by manipulations that interfered with voicing, namely, the *whisper*, *frequency jumps* and *mixed* NLP conditions. In comparison, the impact of *chaos* was moderate and possibly mediated by partially obscuring voicing distinctions when it was introduced on top of loud street noise. Crucially, subharmonics and AM had no negative effect on intelligibility, even when they occurred unpredictably in several episodes and the listening task was made very challenging.

## 4. Discussion

In this series of psychoacoustic experiments, we investigated the impact of irregular phonation caused by nonlinear vocal phenomena (NLP) on speech intelligibility. Apart from its relevance to clinical work on voice pathology, this issue is of central importance to the study of the evolution of language. To claim that derived anatomical features of the human vocal apparatus, such as the loss of vocal membranes, are adaptations for speech, we must show that they make the voice more suitable for conveying phonetic information. Additional supralaryngeal resonators (air sacs) and oscillators (vocal membranes), which are present in all apes except for humans, make the voice source less stable and predisposed to NLP [12–15,39]. Based on this anatomical evidence and biomechanical simulations, Nishimura *et al*. [15] have argued that the disappearance of these structures is adaptive for speech, but they did not test whether NLP actually impede speech intelligibility. In this article, we report two basic findings. First, we demonstrate that vocal instability in the form of sudden jumps in voice pitch and obscured voicing distinctions is indeed detrimental for speech intelligibility. However, continuous forms of NLP, including subharmonics, sidebands and even chaos, have little or no negative impact on speech intelligibility, and might even improve it in certain cases. Indeed, our second main finding is that a dense vocal spectrum caused by subharmonics or AM is actually ideal for highlighting formant transitions, and even the net effect of highly irregular, chaotic phonation is not so clear-cut. Thus, the assumption that NLP intrinsically mask the vocal tract conformation may need to be reconsidered. We discuss the implications of these two results for the evolution of language and vocal communication in modern humans.

First of all, what kind of vocal instability compromises speech perception, and why? According to our findings, sudden frequency jumps or voice breaks greatly reduce the intelligibility of short phrases. Although entire sentences are ecologically valid targets for studying intelligibility, the drawback of using them is that syntactic and semantic cues become very influential [73]. Indeed, speech intelligibility is negatively affected by any linguistically incorrect or misleading intonation [69,70] such as flattened *f*_o_ contours [74]. We also found that frequency jumps were the only NLP type that reduced the listeners’ ability to distinguish between a few target numbers, in which *f*_o_ contours carried no phonetic information, but this effect was very weak. Voice breaks and jumps may affect comprehension because they are simply distracting, particularly when the listening task is challenging. On the other hand, we failed to observe the expected difference between NLP added continuously throughout a sentence or sporadically in multiple episodes, with the exception of repeatedly switching between phonated and whispered sound production, which was harder to understand than ‘whispering’ throughout the entire target phrase.

Thus, it appears that any loss of speech intelligibility arising from the presence of NLP was primarily caused by interfering with voicing—phonetically appropriate voice onsets/offsets and smooth, predictable *f*_o_ contours. In comparison, the quality of phonation was far less important: voice signals could be tonal, biphonated (with subharmonics or AM) or even chaotic, and still the speech retained normal or near-normal intelligibility. In other words, listeners had no problem understanding what was being said even in ‘harsh-sounding’ speech with lots of NLP, but they struggled when speech was marked by sudden changes in pitch and voicing. A clear implication for voice pathology is the need to carefully distinguish between different types of irregularities in voice production. Clearly, audible subharmonics may be relatively common in the speech of healthy speakers [50], but our results show that the resulting vocal hoarseness, though perceptually obvious and perhaps unpleasant, has a negligible effect on intelligibility. In contrast, a shaky voice with frequent voice breaks or jumps can be much more disruptive in producing understandable speech, potentially requiring clinical intervention.

The second key finding is that continuous, rough-sounding NLP may actually help to highlight the formants that would otherwise remain inaudible in high-pitched vocalizations. This has important implications for other forms of vocal communication besides speech. Angry roars and screams have a high *f*_o_ as a byproduct of being loud [36], but the tradeoff is that their spectrum has too few harmonics to excite vocal tract resonances (figure 1A), which means that they may fail to encode filter-related formant information and potentially become less individually distinct [60,61]. If NLP partly compensate for this high *f*_o_ and keep formants audible, while also lowering the apparent pitch and avoiding a ‘tinny’ voice quality [47], they may be highly adaptive for vocal intimidation [36]. While we did not directly investigate shouted speech or singing in the present study, we have shown in past work that shouted speech gives rise to more NLP and is an effective strategy for vocal intimidation [36]. It is a reasonable hypothesis to test in follow-up studies whether the intelligibility of shouted speech at high *f*_o_ can be enhanced by volitionally adding some vocal roughness—for example, by engaging the ventricular folds or the mucosa covering the arytenoids, as in rock singing [44].

The experiments reported here have important limitations related to the method of manipulating NLP and the range of tested stimuli. A complete methodological separation between the source and filter is necessary for a stringent test of the impact of NLP at the source level on the intelligibility of the resulting speech signal. This requirement was satisfied in Experiment 1, which used fully synthetic phonemes, but not in Experiments 2 and 3, in which we took the filter (formants) from original recordings and only manipulated the source (vocal fold dynamics). As a result, NLP manipulations in these experiments would be unlikely to dramatically boost formant detection owing to revealing originally undersampled resonances of the vocal tract. This means that we were more likely to find no effects or detrimental effects of NLP on speech intelligibility in words and sentences than to find that NLP improved speech comprehension, making this a rather conservative test. A promising alternative to explore in follow-up studies would be to use articulatory synthesis to combine the perfect control of Experiment 1 with the ability to create more linguistically complex stimuli, as in Experiments 2 and 3. The second major limitation is the nature of performed NLP manipulations. While subharmonics and AM were computationally and perceptually realistic, the *chaos* condition relied on an imitation of true deterministic chaos [75]. Moreover, NLP in real speech are likely to co-occur with changes in voice intensity, intonation and most importantly with changes in the source spectrum, while here we preserved the original spectral envelopes. Thirdly, we only tested the intelligibility of a single language, English, which heavily relies on phonetic features potentially obscured by some NLP, such as voicing and intonation. Hypothetically, the effect of irregular phonation on intelligibility may be weaker in languages that do not contrast voiced and voiceless consonants (about 1/3 of all languages [76]), but stronger in languages that use lexical tones or creaky/breathy phonemic distinctions. From a functional perspective, if humans still possessed the less-stable vocal source of chimpanzees or lacked perfect control over voice onset, speech might compensate by employing other phonetic contrasts instead, and then whispering or chaos would presumably not interfere with comprehension. In other words, what looks like an anatomical adaptation might just as easily be a case of cultural selection adapting speech to the vocal apparatus, and not the other way around.

In conclusion, our results strongly support the hypothesis that the anatomical simplification of the vocal apparatus in humans is adaptive for speech because it stabilizes the vocal source [15]. In contrast, we do not find evidence that NLP mask formant transitions. Instead, any detrimental effects of NLP are apparently caused by interfering with (i) precisely timed and perceptually salient onsets and offsets of phonation and (ii) smooth intonation contours. Thus, our contribution is to shift the focus from the filter back to the vocal source itself. We also show large differences between NLP types in terms of their impact on speech intelligibility and demonstrate that low-frequency modulation of the kind created by the ventricular folds of anatomically modern humans has the potential to facilitate, not impede, formant perception. Furthermore, NLP do not necessarily represent uncontrollable vocal slips—they can also be seen as desirable design features of the vocal apparatus that can be turned on and off as needed [77], particularly given the incredible control that we humans have over our vocal output [4]. Thus, we argue that the ability to produce NLP is not lost in modern humans, as they remain ubiquitous and functional in human singing [45] and nonverbal communication [47,50]. Rather, we argue that NLP were only brought under better control as part of the suite of anatomical and neurological adaptations for speech.

## Data Availability

Audio recordings, datasets and R scripts for data analysis can be downloaded from [78]. Supplementary material is available online [79].

## References

[1] Bickerton D. 2014 More than nature needs. Cambridge, MA: Harvard University Press.

[2] Fitch WT. 2010 The evolution of language. New York, NY: Cambridge University Press.

[3] Cerkevich CM, Rathelot JA, Strick PL. 2022 Cortical basis for skilled vocalization. Proc. Natl Acad. Sci. USA **119**, e2122345119. (10.1073/pnas.2122345119) 35507879 PMC9171651

[4] Ackermann H, Hage SR, Ziegler W. 2014 Brain mechanisms of acoustic communication in humans and nonhuman primates: An evolutionary perspective. Behav. Brain Sci. **37**, 529–546. (10.1017/s0140525x13003099) 24827156

[5] Fitch WT. 2018 The biology and evolution of speech: a comparative analysis. Annu. Rev. Linguist. **4**, 255–279. (10.1146/annurev-linguistics-011817-045748)

[6] Ekström AG. 2024 Correcting the record: phonetic potential of primate vocal tracts and the legacy of Philip Lieberman (1934−2022). Am. J. Primatol. **86**, e23637. (10.1002/ajp.23637) 38741274

[7] Lieberman P. 1969 Vocal tract limitations on the vowel repertoires of rhesus monkey and other nonhuman primates. Science **164**, 1185–1187. (10.1126/science.164.3884.1185) 4976883

[8] Lieberman P. 2012 Vocal tract anatomy and the neural bases of talking. J. Phon. **40**, 608–622. (10.1016/j.wocn.2012.04.001)

[9] Hewitt G, MacLarnon A, Jones KE. 2002 The functions of laryngeal air sacs in primates: a new hypothesis. Folia Primatol. **73**, 70–94. (10.1159/000064786) 12207055

[10] Nishimura T. 2020 Primate vocal anatomy and physiology: similarities and differences between humans and nonhuman primates. In The origins of language revisited: differentiation from music and the emergence of neurodiversity and autism (ed. N Masataka), pp. 25–53. Singapore: Springer.

[11] Hayama S. 1970 The Saccus laryngis in primates. J. Anthropol. Soc. Nippon **78**, 274–298. (10.1537/ase1911.78.274)

[12] Nakamura K, Kanaya M, Matsushima D, Dunn JC, Hirabayashi H, Sato K, Tokuda IT, Nishimura T. 2024 Twin vocal folds as a novel evolutionary adaptation for vocal communications in lemurs. Sci. Rep. **14**, 3631. (10.1038/s41598-024-54172-z) 38351102 PMC10864409

[13] Inoue T, Shiozawa K, Matsumoto T, Kanaya M, Tokuda IT. 2024 Nonlinear dynamics and chaos in a vocal-ventricular fold system. Chaos **34**, 023134. (10.1063/5.0155215) 38386906

[14] Kanaya M, Matsumoto T, Uemura T, Kawabata R, Nishimura T, Tokuda IT. 2022 Physical modeling of the vocal membranes and their influence on animal voice production. JASA Express Lett **2**, 111201. (10.1121/10.0015071) 36456367

[15] Nishimura T *et al*. 2022 Evolutionary loss of complexity in human vocal anatomy as an adaptation for speech. Science **377**, 760–763. (10.1126/science.abm1574) 35951711

[16] Fant G. 1960 Acoustic theory of speech production: with calculations based on X-ray studies of Russian articulations. The Hague, The Netherlands: Mouton.

[17] Titze I. 2000 Principles of voice production, 2nd printing. Iowa City, IA: National Center for Voice and Speech.

[18] Behrman A. 2021 Speech and voice science, 4th edn. San Diego, CA: Plural publishing.

[19] Johnson K. 2011 Acoustic and auditory phonetics. Chichester, UK: Wiley-Blackwell.

[20] Remez RE, Rubin PE, Pisoni DB, Carrell TD. 1981 Speech perception without traditional speech cues. Science **212**, 947–949. (10.1126/science.7233191) 7233191

[21] Elliott TM, Theunissen FE. 2009 The modulation transfer function for speech intelligibility. PLoS Comput. Biol. **5**, e1000302. (10.1371/journal.pcbi.1000302) 19266016 PMC2639724

[22] Ryalls JH, Lieberman P. 1982 Fundamental frequency and vowel perception. J. Acoust. Soc. Am. **72**, 1631–1634. (10.1121/1.388499) 7175033

[23] Fitch D. T, Anikin A, Pisanski K, Valente D. 2025 Formant analysis of vertebrate vocalizations: achievements, pitfalls & promises. BMC Biology. (10.1186/s12915-025-02188-w) PMC11974057 40189499

[24] Bradlow AR, Torretta GM, Pisoni DB. 1996 Intelligibility of normal speech I: global and fine-grained acoustic-phonetic talker characteristics. Speech Commun. **20**, 255–272. (10.1016/s0167-6393(96)00063-5) 21461127 PMC3066472

[25] Sommers MS, Barcroft J. 2006 Stimulus variability and the phonetic relevance hypothesis: effects of variability in speaking style, fundamental frequency, and speaking rate on spoken word identification. J. Acoust. Soc. Am. **119**, 2406–2416. (10.1121/1.2171836) 16642853

[26] Amano-Kusumoto A, Hosom JP. 2011 A review of research on speech intelligibility and correlations with acoustic features. Report # CSLU-011-001. Portland, OR: Center for Spoken Language Understanding, Oregon Health and Science University (OHSU).

[27] Rostolland D. 1985 Intelligibility of shouted voice. Acta Acust. United Acust. **57**, 103–121.

[28] Charlton BD, Taylor AM, Reby D. 2017 Function and evolution of vibrato-like frequency modulation in mammals. Curr. Biol. **27**, 2692–2697.(10.1016/j.cub.2017.07.046) 28844642

[29] Porcaro CK, Evitts PM, King N, Hood C, Campbell E, White L, Veraguas J. 2020 Effect of Dysphonia and Cognitive-Perceptual Listener Strategies on Speech Intelligibility. J. Voice **34**, 806. (10.1016/j.jvoice.2019.03.013) 31031103

[30] Shen J, Heller Murray E, Kulick ER. 2023 The effect of breathy vocal quality on speech intelligibility and listening effort in background noise. Trends Hear **27**, 1–14. (10.1177/23312165231206925) PMC10566269 37817666

[31] Kallail KJ, Emanuel FW. 1984 An acoustic comparison of isolated whispered and phonated vowel samples produced by adult male subjects. J. Phon. **12**, 175–186. (10.1016/s0095-4470(19)30864-2) 6738036

[32] Tartter VC. 1991 Identifiability of vowels and speakers from whispered syllables. Percept. Psychophys. **49**, 365–372. (10.3758/bf03205994) 2030934

[33] Konno H, Sato R, Imai H, Kudo M. 2014 Deterioration of intelligibility in whispered Japanese speech. In 2014 Asia-Pacific Signal and Information Processing Association Annual Summit and Conference (APSIPA), Chiang Mai, Thailand, pp. 1–4. IEEE. (10.1109/APSIPA.2014.7041723)

[34] Jovičić S. 1998 Formant feature differences between whispered and voiced sustained vowels. Acta Acust. United Acust. **84**, 739–743.

[35] Nathwani K, Richard G, David B, Prablanc P, Roussarie V. 2017 Speech intelligibility improvement in car noise environment by voice transformation. Speech Commun. **91**, 17–27. (10.1016/j.specom.2017.04.007)

[36] Anikin A, Valente D, Pisanski K, Cornec C, Bryant GA, Reby D. 2024 The role of loudness in vocal intimidation. J. Exp. Psychol. **153**, 511–530. (10.1037/xge0001508) 38010781

[37] Herbst CT, Nishimura T, Garcia M, Migimatsu K, Tokuda IT. 2021 Effect of ventricular folds on vocalization fundamental frequency in domestic pigs (Sus scrofa domesticus). J. Voice **35**, 805.e1–805.e15. (10.1016/j.jvoice.2020.01.013) 33388229

[38] de Boer B. 2009 Acoustic analysis of primate air sacs and their effect on vocalization. J. Acoust. Soc. Am. **126**, 3329–3343. (10.1121/1.3257544) 20000947

[39] De Boer B. 2012 Air sacs and vocal fold vibration: implications for evolution of speech. Theoria et Historia Scientiarum **9**, 13–28. (10.12775/v10235-011-0002-5)

[40] Maxfield L, Palaparthi A, Titze I. 2017 New evidence that nonlinear source-filter coupling affects harmonic intensity and fo stability during instances of harmonics crossing formants. J. Voice **31**, 149–156. (10.1016/j.jvoice.2016.04.010) 27501922 PMC5292312

[41] Titze IR. 2008 Nonlinear source-filter coupling in phonation: Theory. J. Acoust. Soc. Am. **123**, 1902–1915. (10.1121/1.2832337) 18529191 PMC2811547

[42] Tokuda IT, Zemke M, Kob M, Herzel H. 2010 Biomechanical modeling of register transitions and the role of vocal tract resonators. J. Acoust. Soc. Am. **127**, 1528–1536. (10.1121/1.3299201) 20329853

[43] Zhang Z, Neubauer J, Berry DA. 2006 The influence of subglottal acoustics on laboratory models of phonation. J. Acoust. Soc. Am. **120**, 1558–1569. (10.1121/1.2225682) 17004478

[44] Borch DZ, Sundberg J, Lindestad PÅ, Thalén M. 2004 Vocal fold vibration and voice source aperiodicity in ‘dist’ tones: a study of a timbral ornament in rock singing. Logop. Phoniatr. Vocology **29**, 147–153. (10.1080/14015430410016073) 15764208

[45] Neubauer J, Edgerton M, Herzel H. 2004 Nonlinear phenomena in contemporary vocal music. J. Voice **18**, 1–12. (10.1016/s0892-1997(03)00073-0) 15070219

[46] Sakakibara KI, Fuks L, Imagawa H, Tayama N. 2004 Growl voice in ethnic and pop styles. In Proc. Int. Symp. on Musical Acoustics, Nara, Japan. http://www.hoku-iryo-u.ac.jp/~kis/paper/isma04.pdf (accessed 3 April 2004).

[47] Anikin A, Pisanski K, Massenet M, Reby D. 2021 Harsh is large: nonlinear vocal phenomena lower voice pitch and exaggerate body size. Proc. R. Soc. B **288**, 20210872. (10.1098/rspb.2021.0872) PMC8261225 34229494

[48] Mende W, Herzel H, Wermke K. 1990 Bifurcations and chaos in newborn infant cries. Phys. Lett. **145**, 418–424. (10.1016/0375-9601(90)90305-8)

[49] Valente D, Magnard C, Koutseff A, Patural H, Chauleur C, Reby D, Pisanski K. 2024 Vocal communication and perception of pain in childbirth vocalizations. Phil. Trans. R. Soc. B **380**, 20240009. (10.1098/rstb.2024.0009) PMC11966154 40176506

[50] Anikin A, Canessa-Pollard V, Pisanski K, Massenet M, Reby D. 2023 Beyond speech: exploring diversity in the human voice. iScience **26**, 108204. (10.1016/j.isci.2023.108204) 37908309 PMC10613903

[51] Bailly L, Henrich N, Pelorson X. 2010 Vocal fold and ventricular fold vibration in period-doubling phonation: Physiological description and aerodynamic modeling. J. Acoust. Soc. Am. **127**, 3212–3222. (10.1121/1.3365220) 21117769

[52] Fuks L, Hammarberg B, Sundberg J. 1998 A self-sustained vocal-ventricular phonation mode: acoustical, aerodynamic and glottographic evidences. KTH TMH QPSR **3**, 49–59.

[53] Bender BK, Cannito MP, Murry T, Woodson GE. 2004 Speech intelligibility in severe adductor spasmodic dysphonia. J. Speech Lang. Hear. Res. **47**, 21–32. (10.1044/1092-4388(2004/003) 15072525

[54] Bottalico P, Murgia S, Puglisi GE, Astolfi A, Ishikawa K. 2021 Intelligibility of dysphonic speech in auralized classrooms. J. Acoust. Soc. Am. **150**, 2912–2920. (10.1121/10.0006741) 34717474

[55] Evitts PM, Starmer H, Teets K, Montgomery C, Calhoun L, Schulze A, MacKenzie J, Adams L. 2016 The impact of dysphonic voices on healthy listeners: listener reaction times, speech intelligibility, and listener comprehension. Am. J. Speech Lang. Pathol. **25**, 561–575. (10.1044/2016_ajslp-14-0183) 27784031

[56] Oliveira GMGF Mrs, de Melo DC Mrs, Serra LSM Dra, Granjeiro RC Dr, Sampaio ALL Dr. 2024 Dysphonia interference in schoolteachers’ speech intelligibility in the classroom. J. Voice **38**, 316–324. (10.1016/j.jvoice.2021.09.004) 34772594

[57] Schiller IS, Morsomme D, Kob M, Remacle A. 2020 Noise and a speaker’s impaired voice quality disrupt spoken language processing in school-aged children: evidence from performance and response time measures. J. Speech Lang. Hear. Res. **63**, 2115–2131. (10.1044/2020_jslhr-19-00348) 32569506

[58] von Lochow H, Lyberg-Åhlander V, Sahlén B, Kastberg T, Brännström KJ. 2018 The effect of voice quality and competing speakers in a passage comprehension task: perceived effort in relation to cognitive functioning and performance in children with normal hearing. Logop. Phoniatr. Vocology **43**, 32–41. (10.1080/14015439.2017.1307446) 28367655

[59] Fitch WT, Fritz JB. 2006 Rhesus macaques spontaneously perceive formants in conspecific vocalizations. J. Acoust. Soc. Am. **120**, 2132–2141. (10.1121/1.2258499) 17069311

[60] Rendall D, Owren MJ, Rodman PS. 1998 The role of vocal tract filtering in identity cueing in rhesus monkey (Macaca mulatta) vocalizations. J. Acoust. Soc. Am. **103**, 602–614. (10.1121/1.421104) 9440345

[61] Owren MJ, Rendall D. 2003 Salience of Caller Identity in Rhesus Monkey (Macaca mulatta) Coos and Screams: Perceptual Experiments With Human (Homo sapiens) Listeners. J. Comp. Psychol. **117**, 380–390. (10.1037/0735-7036.117.4.380) 14717639

[62] Guasch O, Freixes M, Arnela M, Van Hirtum A. 2024 Controlling chaotic vocal fold oscillations in the numerical production of vowel sounds. Chaos Solitons Fractals **182**, 114740. (10.1016/j.chaos.2024.114740)

[63] Fitch WT, Neubauer J, Herzel H. 2002 Calls out of chaos: the adaptive significance of nonlinear phenomena in mammalian vocal production. Anim. Behav. **63**, 407–418. (10.1006/anbe.2001.1912)

[64] Pisanski K, Fraccaro PJ, Tigue CC, O’Connor JJM, Feinberg DR. 2014 Return to Oz: voice pitch facilitates assessments of men’s body size. J. Exp. Psychol. **40**, 1316–1331. (10.1037/a0036956) 24933617

[65] Anikin A. 2019 Soundgen: An open-source tool for synthesizing nonverbal vocalizations. Behav. Res. Methods **51**, 778–792. (10.3758/s13428-018-1095-7) 30054898 PMC6478631

[66] Bürkner PC. 2017 brms: An R package for bayesian multilevel models using stan. J. Stat. Softw **80**, 1–28. (10.18637/jss.v080.i01)

[67] Pisanski K, Bryant GA, Cornec C, Anikin A, Reby D. 2022 Form follows function in human nonverbal vocalisations. Ethol. Ecol. Evol. **34**, 303–321. (10.1080/03949370.2022.2026482)

[68] Rothauser E. 1969 IEEE recommended practice for speech quality measurements. IEEE Trans. Audio Electroacoustics **17**, 225–246. (10.1109/TAU.1969.1162058)

[69] Binns C, Culling JF. 2007 The role of fundamental frequency contours in the perception of speech against interfering speech. J. Acoust. Soc. Am. **122**, 1765–1776. (10.1121/1.2751394) 17927436

[70] Miller SE, Schlauch RS, Watson PJ. 2010 The effects of fundamental frequency contour manipulations on speech intelligibility in background noise. J. Acoust. Soc. Am. **128**, 435–443. (10.1121/1.3397384) 20649237

[71] Chen SF, Beeferman D, Rosenfeld R. 1998 Evaluation metrics for language models. In Proceedings of the DARPA Broadcast News Transcription and Understanding Workshop, pp. 275–280. Pittsburgh, PA: Carnegie Mellon University. (10.1184/R1/6605324.V1)

[72] Ishikawa K, Nudelman C, Park S, Ketring C. 2021 Perception and acoustic studies of vowel intelligibility in dysphonic speech. J. Voice **35**, 659.e11–659.e24. (10.1016/j.jvoice.2019.12.022) 31952898

[73] Miller N. 2013 Measuring up to speech intelligibility. Int. J. Lang. Commun. Disord. **48**, 601–612. (10.1111/1460-6984.12061) 24119170

[74] Watson PJ, Schlauch RS. 2008 The effect of fundamental frequency on the intelligibility of speech with flattened intonation contours. Am. J. Speech Lang. Pathol. **17**, 348–355. (10.1044/1058-0360(2008/07-0048)) 18840699

[75] Anikin A, Herbst C. 2025 How to analyze and manipulate nonlinear phenomena in voice recordings. Phil. Trans. R. Soc. B **380**, 20240003. (10.1098/rstb.2024.0003) PMC11966163 40176526

[76] Maddieson I. 2013 Voicing in Plosives and Fricatives (v2020.3). In The world atlas of language structures online. Leipzig, Germany: Max Planck Institute for Evolutionary Anthropology. (10.5281/zenodo.7385533)

[77] Rendall D. 2025 Nonlinear phenomena in animal vocalizations: do they reflect alternative functional modes of voice control, or ‘leaked’ cues to quality or condition, or both? Phil.Trans. R. Soc. B **380**, 20240010. (10.1098/rstb.2024.0010) PMC11966166 40176519

[78] Anikin A, Pisanski K, Reby D. NLP and speech intelligibility. (10.17605/OSF.IO/TK4SB) PMC11966171 40176514

[79] Anikin A, Reby D, Pisanski K. 2025 Supplementary material from: Nonlinear vocal phenomena and speech intelligibility. Figshare. (10.6084/m9.figshare.c.7708734) PMC11966171 40176514

